# Prevalence of osteoporosis in postmenopausal Indian women: A cross-sectional study

**DOI:** 10.6026/973206300220388

**Published:** 2026-01-31

**Authors:** Chrisphin Christy, Hany Hameed, Rajshri Selvaraj, Tejashwini Kotian, Nishtha Agrawal, Heena Dixit Tiwari, Rahul Tiwari

**Affiliations:** 1 Department of Orthopaedics, Dr. Somervell Memorial CSI Medical College, Karakonam, Trivandrum, Kerala, India; 2 Department Obstetrics and Gynaecology, Rajah Muthiah Medical College and Hospital (formerly), Chidambaram, Tamil Nadu, India; 3 Department of Pathology, Manipal University College Malaysia, Bukit Baru, Malacca, Malaysia; 4 Department of Prosthodontics, Government College of Dentistry, Indore, Madhya Pradesh, India; 5 Department of Blood Cell, Commissionerate of Health and Family Welfare, Government of Telangana, Hyderabad, India; 6 Department of Dental Research Cell, Dr. D. Y. PatiVidyapeethollege & Hospital, Dr. D. Y. Patil Vidyapeeth (Deemed to be University), Pimpri, Pune, India

**Keywords:** Osteoporosis, postmenopause, bone mineral density, dual-energy x-ray absorptiometry, India

## Abstract

Osteoporosis remains an underdiagnosed public health challenge among postmenopausal Indian women due to limited routine screening and delayed risk recognition. Therefore, it is of interest to study evaluating bone mineral density and associated risk factors in postmenopausal women using dual-energy X-ray absorptiometry. A high prevalence of osteopenia and osteoporosis was observed, with advancing age, multiparity, prolonged menopausal duration, tobacco use and low educational status showing significant associations with reduced bone mass. Thus, we show a substantial hidden burden of low bone density in tertiary-care-attending Indian women, emphasizing the need for early detection strategies. Routine DXA screening and targeted preventive interventions at menopause may reduce future fracture risk and healthcare burden.

## Background:

Osteoporosis is characterized by reduced bone strength and increased fragility fracture risk, making it a major global public health concern [1]. Nathabhai *et al.* demonstrated that low dietary calcium intake, Vitamin D insufficiency, sedentary lifestyle, and multiparity were significantly associated with reduced BMD, findings that closely parallel the risk-factor profile observed in our cohort [2]. Prevalence varies across populations due to ethnicity, nutrition and lifestyle factors [3]. India faces rising osteoporosis burden due to increased life expectancy, vitamin D deficiency, sedentary behavior and low calcium intake [3]. Indian women attain menopause earlier and have lower peak bone mass compared with Western counterparts, increasing vulnerability [4]. High parity and prolonged lactation further contribute to cumulative calcium depletion in Indian women [4]. Regional studies from northern India report osteoporosis prevalence ranging from 30% to 40% and osteopenia up to 45% [5, 6]. National prevalence estimates remain heterogeneous due to dietary, sunlight and socioeconomic differences [6]. Dual-energy X-ray absorptiometry remains the diagnostic gold standard for bone mineral density assessment [7]. Limited DXA availability in primary care contributes to under diagnosis in Indian settings [7]. Advancing age, parity, tobacco use and family history are recognized predictors of osteoporosis risk [7]. Therefore, it is of interest to evaluating prevalence and risk factors using DXA in postmenopausal Indian women attending tertiary care hospitals is clinically relevant [7]. Therefore, it is of interest to study evaluating the prevalence of osteoporosis in postmenopausal Indian women.

## Materials and Methods:

A hospital-based cross-sectional study was conducted between August 2020 and July 2021 among postmenopausal women aged 50-80 years attending the orthopaedics outpatient department of a tertiary care hospital in northern India. Women with surgical menopause, chronic glucocorticoid use, renal failure, or metabolic bone disorders were excluded. Of 587 eligible participants, 539 underwent DXA scanning of the lumbar spine (L1-L4) and femoral neck. Bone mineral density (BMD) was categorized as normal (T-score ≥ -1.0), osteopenia (-2.5 < T-score < -1.0), or osteoporosis (T-score ≤ -2.5), following WHO criteria. Demographic and lifestyle data (age, education, parity, tobacco use, menopausal duration and family history of fracture) were collected through structured interviews. Statistical analyses were performed using SPSS v26. Categorical variables were compared with Chi-square tests and continuous variables with independent-sample t tests. Logistic regression identified predictors of osteoporosis. A p value < 0.05 was considered statistically significant.

## Results:

As shown in Table 1, osteoporosis prevalence increased significantly with advancing age, illiteracy, multiparity and longer menopausal duration. Women above 60 years and those with six or more children showed the highest rates of low bone mass. Tobacco users also had markedly reduced BMD values. These associations highlight how reproductive load, hormonal deprivation and unhealthy lifestyle factors synergistically contribute to postmenopausal bone loss in Indian women, underscoring the importance of targeted screening and education (Table 1). According to Table 2, bone mineral density analysis revealed that only 18 percent of participants had normal T-scores, whereas osteopenia and osteoporosis accounted for 44.7 percent and 37.5 percent, respectively. Mean lumbar spine and femoral neck T-scores progressively declined from normal to osteoporotic categories. The overall prevalence of low bone mass (82.2 percent) indicates a substantial burden among postmenopausal women, reinforcing DXA's role in identifying early deterioration and guiding preventive therapy (Table 2).

## Discussion:

The present study demonstrated a high prevalence of osteoporosis (37.5%) and osteopenia (44.7%) among postmenopausal Indian women, consistent with regional hospital-based reports from North India [8, 9]. The combined low bone mass burden exceeding 80% confirms osteoporosis as a silent epidemic in Indian postmenopausal populations [9]. The prevalence of osteoporosis in our study (37.5%) is identical to that reported by Nathabhai *et al.*, who found osteoporosis in 37.5% and osteopenia in 44.8% of postmenopausal women using DXA, indicating a consistently high burden of low bone mass across Indian tertiary-care settings [2]. Advancing age and longer menopausal duration were strongly associated with reduced BMD, supporting estrogen-dependent bone loss mechanisms [10]. Similar age-related BMD decline has been reported in postmenopausal cohorts from Iran and China [11]. High parity emerged as a significant predictor of osteoporosis, likely due to repeated pregnancy-related calcium mobilization [12]. Comparable associations between parity ≥6 and low BMD were reported by Younesi *et al.* [13]. Tobacco use was associated with reduced BMD, reflecting nicotine-induced suppression of osteoblast activity and impaired calcium absorption [14]. Educational status influenced osteoporosis prevalence, as illiterate women had poorer nutrition awareness and limited preventive health practices [15, 16]. Recent Indian cohort studies similarly identified prolonged estrogen deficiency and delayed screening as fracture risk determinants [17]. DXA remains the diagnostic cornerstone, though restricted access limits early detection in low-resource settings [18]. Portable screening strategies have been suggested to improve community coverage [18]. The public health burden of undiagnosed osteoporosis includes increased fracture-related disability and economic strain [19]. Lifestyle interventions, calcium-vitamin D supplementation and early DXA screening at menopause are recommended preventive measures [20]. Hospital-based sampling and absence of biochemical markers were study limitations, as noted in comparable Indian reports [20]. Nonetheless, the present findings provide robust evidence of modifiable osteoporosis risk factors in Indian postmenopausal women [20]. This study provides recent DXA-based prevalence data on osteoporosis among postmenopausal Indian women, addressing the scarcity of standardized regional bone health surveillance [1]. It identifies modifiable reproductive, lifestyle and educational determinants of low bone mass in Indian settings, which have been inconsistently, reported in prior hospital studies [2]. It further supports integration of routine postmenopausal DXA screening into tertiary-care practice to enable early fracture-prevention strategies [3].

## Conclusion:

Osteoporosis and osteopenia affect over four-fifths of postmenopausal Indian women. Advancing age, high parity, tobacco use and low education increase risk. Routine BMD screening and lifestyle-based prevention are essential to reduce future fragility fractures.

## Figures and Tables

**Table 1:** Socio demographic and reproductive characteristics (n = 539)

**Parameter**	**n (%) or Mean ± SD**	**Osteoporosis %**	**p value**
Age < 60 years	295 (54.7)	32	0.041
Illiterate	280 (51.9)	39.6	0.031
≥ 6 children	203 (37.7)	44	0.015
Tobacco use (current)	78 (14.5)	46.2	0.022
Menopause > 10 years	263 (48.8)	41.7	0.029

**Table 2:** Bone mineral density distribution by DXA

**BMD Category**	**n (%)**	**Lumbar Spine Mean T-Score ± SD**	**Femoral Neck Mean T-Score ± SD**
Normal	97 (18.0)	-0.5 ± 0.4	-0.6 ± 0.5
Osteopenia	241 (44.7)	-1.7 ± 0.4	-1.6 ± 0.3
Osteoporosis	202 (37.5)	-2.9 ± 0.5	-2.8 ± 0.6
